# A Principal Component Informed Approach to Address Polygenic Risk Score Transferability Across European Cohorts

**DOI:** 10.3389/fgene.2022.899523

**Published:** 2022-07-18

**Authors:** Katri Pärna, Ilja M. Nolte, Harold Snieder, Krista Fischer, Estonian Biobank Research Team, Davide Marnetto, Luca Pagani

**Affiliations:** ^1^ Institute of Genomics, University of Tartu, Tartu, Estonia; ^2^ Department of Epidemiology, University of Groningen, Groningen, Netherlands; ^3^ Institute of Mathematics and Statistics, University of Tartu, Tartu, Estonia; ^4^ Department of Neurosciences “Rita Levi Montalcini”, University of Turin, Torino, Italy; ^5^ Department of Biology, University of Padova, Padova, Italy

**Keywords:** genome-wide association study, population structure, principal component analysis, polygenic risk score, transferability

## Abstract

One important confounder in genome-wide association studies (GWASs) is population genetic structure, which may generate spurious associations if not properly accounted for. This may ultimately result in a biased polygenic risk score (PRS) prediction, especially when applied to another population. To explore this matter, we focused on principal component analysis (PCA) and asked whether a population genetics informed strategy focused on PCs derived from an external reference population helps in mitigating this PRS transferability issue. Throughout the study, we used two complex model traits, height and body mass index, and samples from UK and Estonian Biobanks. We aimed to investigate 1) whether using a reference population (1000G) for computation of the PCs adjusted for in the discovery cohort improves the resulting PRS performance in a target set from another population and 2) whether adjusting the validation model for PCs is required at all. Our results showed that any other set of PCs performed worse than the one computed on samples from the same population as the discovery dataset. Furthermore, we show that PC correction in GWAS cannot prevent residual population structure information in the PRS, also for non-structured traits. Therefore, we confirm the utility of PC correction in the validation model when the investigated trait shows an actual correlation with population genetic structure, to account for the residual confounding effect when evaluating the predictive value of PRS.

## Introduction

The last 15 years have offered great opportunities to explore the genetic component of complex diseases and traits by using genome-wide association studies (GWASs) (Visscher et al., 2017). Associated variants generally have a small effect on the biological outcome (Wray et al., 2007; Visscher et al., 2017) and are often combined into a polygenic risk score (PRS) to estimate a person’s genetic susceptibility for a trait or disease (Wray et al., 2014). PRSs have already demonstrated their clinical potential by detecting individuals in high-risk groups for several diseases such as type 2 diabetes, cardiovascular diseases, Alzheimer’s disease, breast cancer, prostate cancer, and colorectal cancer (Lecarpentier et al., 2017; Khera et al., 2018; Schumacher et al., 2018; Läll et al., 2019; Pärna et al., 2020) sometimes reaching risk detection equal to monogenic mutations (Khera et al., 2018).

Although the concepts of GWAS and PRS are widely used, one important confounder remaining is population genetic structure, which might result in spurious disease associations if not properly accounted for (Helgason et al., 2005; Kang et al., 2010; Choi et al., 2020) and which may hinder the applicability of effect sizes discovered in one cohort to compute PRS in another. Indeed, it has been shown that GWAS summary statistics based on one population might result in a much lower PRS predictability when applied to a population with different structure, that is, limiting its transferability (Duncan et al., 2019; Martin et al., 2019; Bitarello and Mathieson, 2020; Marnetto et al., 2020; Sakaue et al., 2020). For example, Sakaue et al. (2020) detected substructures and differences in PRS performance between these sub-groups among the Japanese population. In particular, it has been shown that the presence of genetic structure in Europe at a continental (Novembre et al., 2008; Peter et al., 2020) and finer geographical scale can bias GWAS-based statistics and affect PRS transferability even between populations with relatively similar genetic backgrounds (Haworth et al., 2019; Kerminen et al., 2019; Sohail et al., 2019; Byrne et al., 2020; Pankratov et al., 2020).

Several methods to control for population genetic structure have been proposed and successfully applied to improve discovery of true genetic effect sizes such as principal component analysis (PCA) (Price et al., 2006), genomic control (GC) (Devlin and Roeder, 1999), linear mixed models (LMMs) (Loh et al., 2015), and linkage disequilibrium score regression (LDSC) (Bulik-Sullivan and Neale, 2015). However, it remains unclear to what extent the correction applied on the discovery cohort may affect the transferability of the resulting summary statistics. Notably, in case of discovery and target set similarity, a contribution of indirect factors other than direct genetic effects would lead to higher PRS prediction accuracy, but likely at a transferability cost, even between groups of the same ancestry (Mostafavi et al., 2020). Here, we focus on correction for population genetic structure *via* PCA, by far the most broadly adopted control method in genetic association studies, where the analysis of each genetic variant in the GWAS is adjusted for the discovery cohort’s specific principal components (PCs) (Price et al., 2006). Despite its broad adoption, as demonstrated by recent analyses (Berg et al., 2019; Sohail et al., 2019; Zaidi and Mathieson, 2020), its efficacy and potential side effects such as the risk of removing part of the phenotype-genotype association along with the population structure are still a matter of discussion. It has been shown, for example, that when the population exhibits recent changes in its genetic structure, the PCs received based on common variants will not capture well the full extent of information and such incomplete correction at each locus could be amplified by summing single SNP effect sizes as done for PRS construction (Mathieson and McVean, 2012; Lawson et al., 2020; Zaidi and Mathieson, 2020). Likewise, GWAS results deriving from large consortia such as GIANT have been shown to still carry residual population stratification, despite PCA correction in the original studies (Berg et al., 2019). In addition, there is still a lack of consensus on whether PC adjustment should be applied only to the discovery or also to the target cohort (Pärna et al., 2020; Choi et al., 2020; Läll et al., 2017; Abdellaoui et al., 2019; Privé et al., 2022; Wünnemann et al., 2019).

It is important to stress that PCs used in such adjustments, both during discovery and testing, are inherently dataset-specific and therefore might introduce cohort-specific biases that limit PRS transferability. We hypothesized that a broader population dataset to receive the PCs to adjust for in the discovery cohort could mitigate these cohort-specific biases, hence decreasing the summary statistics transferability issues and counterbalancing the lower prediction accuracy of the resulting PRS performance when applied in another cohort. This could be achieved by projecting the samples onto a reference PC space, as previously done for very large discovery sets (Bycroft et al., 2018). Therefore, here, we set out to systematically investigate whether 1) decreasing the specificity of the PCs used to correct for population structure in the discovery cohort may improve the model fit of the resulting PRS, when applied to a cohort from a population different from the one used for the discovery and 2) whether or not adding PCs in the validation model (whether or not specific to the validation/target cohort) increases the model fit in the target set.

We adopted two quantitative model traits, height, and body mass index (BMI), each with its peculiar dependence on population stratification. We computed GWAS summary statistics in one European cohort (United Kingdom Biobank, UKBB) for the calculations of PRS and validated these in independent subsets from the same cohort (UKBBtest) and from another European cohort (Estonian Biobank, EstBB).

Although the PC projection approach presented here presumably leads to an increase in false positives when discovering new GWAS loci, we consider the projection approach useful in testing the PRS prediction performance. Our exploration is indeed intended to inform the best strategy to adopt when applying publicly available effect sizes onto individuals coming from populations for which available sample size is not sufficient to perform independent discovery.

## Methods

### Study Populations

Genetic data from the UK Biobank (UKBB) (Bycroft et al., 2018), Estonian Biobank (EstBB) (Leitsalu et al., 2015) and 1000 Genomes Project (1000G) phase 3 were used for the current study (Auton et al., 2015). UKBB and EstBB have been approved by the North West Centre for Research Ethics Committee (11/NW/0382) and by the Ethics Committee of Human Studies, University of Tartu, Estonia, respectively, and all participants have signed an informed consent. We selected 362,846 unrelated individuals with European ancestry from UKBB. To define the genetically “European” sample, we adapted a method from the Neale Lab (https://github.com/Nealelab/UK_Biobank_GWAS) to select samples which were closer than 7 standard deviations cumulated over the first 6 PCs pre-computed by the UKBB workgroup with respect to the UKBB samples used for GWAS in previous studies (Bycroft et al., 2018). Second, we removed up to third-degree relatives. We divided the UKBB data in 3 independent sets: (1) a discovery set (UKBBtrain) with 350,745 individuals, (2) a target set (UKBBtest) with 7,100 individuals, and (3) an external group to build PC space onto which the other samples were projected (*n* = 5,000). Such a sample subdivision has been devised to maximize the discovery set following what is considered the golden standard for GWAS (Marees et al. (2017)) (Marees et al., 2018). From the EstBB, after removing up to third-degree relatives as in the UKBB dataset, we randomly selected a target set (EstBBtest; *n* = 7,070) and an external group to build a PC space (*n* = 5,000). The 1000G phase 3 (*n* = 2,504) genetic dataset was used as an external publicly available reference for building a PC space.

### Genetic Data Filtering

We started with the set of 784,256 autosomal SNPs genotyped in the UKBB with the UK Axiom Array by Affymetrix (Affymetrix, 2015), which were extracted from each study sample: (1) UKBBtrain, (2) UKBBtest, (3) external UKBB sample, (4) EstBBtest, (5) external EstBB sample, and (6) 1000G. On genetic data of each study sample, we applied the following quality control steps: removing duplicates, indels and palindromic SNPs, ≤ 5% missing data allowed and removing SNPs with minor allele frequency less than 0.01. After the filtering steps, we had *n* = 557,215, *n* = 556, 834 and *n* = 529, 030 SNPs left for the further analysis in UKBBtrain, UKBBtest, and EstBBtest, respectively.

### Principal Component Analysis

Four different PC spaces were built with different sets of individuals used to infer the eigenvectors (1) PC_UKBB_ or PC_EstBB_ include the 5,000 external individuals from the cohort depending on whether the analysis is run on UKBB or EstBB, respectively, (2) PC_1KG_ includes all samples from 1000G (*n* = 2,504); (3) PC_EUR_ includes the European samples from 1000G (*n* = 503); and (4) PC_NEU_ includes non-European samples from 1000G (*n* = 2,001). For all PC spaces listed above, the individuals from the discovery and target sets, which were independent of the ones used to infer the PCA eigenvectors, were projected onto the generated PC space to obtain their PC coordinates. The PCAs were conducted with Eigensoft-6.1.4 software (Price et al., 2006) each time performing LD pruning on the relevant dataset using the parameters *--indep-pairwise 50 10 0.1*. Outlier individuals (>6 SD along one or more of the top 10 PCs of each experiment) were removed during five iterations of PC analyses. Least square optimization was applied for interpolation (projection) of the remaining samples onto the four PC spaces. Specific to each PCA, with the --*poplistname* and --*indivname* parameters, a subset of individuals was selected to compute the PC space. We also performed additional PCAs to explore the impact of 1) size of the sample sets used to compute the PCs—we ran the above by a fixed sample size of 500 samples; 2) effect of shrinkage on the PC projection (run PCs with *shrinkmode*: YES); 3) use identity by descent (IBD) matrix instead of raw genotypes to compute a Multidimensional Scaling (MDS) and compared it with the genotype-based PCAs.

### GWASs for Height and BMI

GWASs for height and BMI were performed based on the UKBBtrain data (*n* = 350,745 individuals and *n* = 557,215 SNPs left after the quality control steps). Assuming an additive genetic model, summary statistics were estimated with PLINK version-1.9.0 (Purcell et al., 2007) using a linear regression analysis adjusted for age, sex, genotyping platform, and, except for the control model, 20 principal components Eq. 1.

$$\hat{trait}={\hat{\beta }}_{0}+{\hat{\beta }}_{1}age+{\hat{\beta }}_{2}sex+{\hat{\beta }}_{3}gp+{\hat{\beta }}_{4}X+{\hat{\beta }}_{5}PC1+{\hat{\beta }}_{6}PC2+\ldots +{\hat{\beta }}_{24}PC20+\varepsilon_{i}$$

Eq. 1 was applied in all GWASs, except the control one, where no PC adjustment was used. Trait: BMI or height; gp = genotyping platform; X = SNP; PC = principal component; $\varepsilon_{i}$ = random error term.

For both traits, five different GWASs were performed: a control GWAS with no PC adjustment plus four GWASs each adjusted for one of the four PC sets derived as described in the section principal component analysis.

### PRS Calculation and Testing

The summary statistics from the five GWASs described above were next used for PRS calculation in two independent target sets (UKBBtest with *n* = 556,834 SNPs and EstBBtest with *n* = 529,030 SNPs); PRSs were computed as a sum of risk variants that were more significant than a prespecified threshold (see below) weighted by the effect sizes from the GWASs. To include only independent SNPs in the PRS, clumping was applied with the parameters: -*clump-r2 0.05 --clump-p 1 --clump-kb 1000* using PLINK version-1.9.0. To select the best-performing set of SNPs for PRS, we applied different *p*-value cut-offs (0.00005, 0.0005, 0.001, 0.005, 0.01, 0.05, 0.1, and 0.5) from which PRSice version 2.2.11. b (Choi et al., 2020) flags the best-performing *p*-value threshold resulting in the PRS with the highest *R* ^2^ value. PRS was standardized for better interpretation. Note that since PRSs are constructed based on different GWASs, across the different validation models, the best-performing PRS can contain different numbers of SNPs.

To assess the association between the outcome trait and a PRS, we fitted a linear regression model on the target sets of the UKBBtest and EstBBtest, including the PRS and the covariates age, sex, genotyping platform/batches and, except for a control model, 20 PCs. The five PRS defined above were independently tested in combination with each one of the five different sets of PCs (as defined in the “Principal component analysis” section) or no PCs for the control model (five options), yielding 25 different validation models. When analyzing the UKBBtest and EstBBtest cohorts, PCs were derived either from the same PC spaces constructed from the 1000G data (PC spaces 2–4) or from the one with the 5,000 external individuals from UKBB or EstBB, accordingly.

### PRS, PC, and Trait Correlations

To investigate the relationships of the traits with PRS and PCs in more detail, we analyzed six different regression models:

(1) trait_res ∼ PCs
(2) PRS ∼ PCs
(3) trait_res ∼ PRS
(4) trait_res ∼ PCs + PRS
(5) trait_res_PRS ∼ PCs
(6) trait_res_PCs ∼ PRS

In these models, for both traits, we used their residuals (trait_res) after first regressing out the effect of non-genetic covariates: age, sex, and genotyping batch. In models 5 and 6, we additionally regressed out either the effect of the standardized PRS or of the first 20 PCs, which we defined as “trait_res_PRS” and “trait_res_PCs”, respectively. We repeated this analysis for each of the five PRSs, while PCs always represented the first 20 dataset-specific principal components (PC_UKBB_ or PC_EstBB_).

To find out if any of these above-mentioned linear regression models provide better fit to our data than the model without independent variables, that is, only with the intercept, we applied the F-test. For the model to be significantly better than the model only with the intercept while accounting for multiple testing, we considered a Bonferroni-corrected one-sided *p*-value cut-off of < 0.005 due to the 10 combinations of PRSs and traits. We used *R* ^2^ to describe how much of the total variance the independent variables in each above-mentioned model could explain for the dependent variable.

### Model Performance

To evaluate model performance, we used the Bayesian Information Criterion (BIC), total *R* ^2^ and added *R* ^2^ by PRS alone. BIC is a criterion for choosing the best-fitting validation model while penalizing for the number of parameters included (Kass and Raftery, 1995; Fabozzi et al., 2014):

BIC=−2likelihood+k ∗ log(n)

where *k* = number of parameters and *n* = number of samples. The lower the BIC value, the better the goodness of fit of the model is. We calculated ΔBIC, the difference between the BIC value for each model minus the BIC of the best fitting model. For ΔBIC, the rules of thumb are (Fabozzi et al., 2014) that a difference of:

a) less than 6 units is considered weak
b) between 6 and 10 is considered strong
c) greater than 10 is considered as a very strong difference in model performance.

*R* ^2^, on the other hand, yields a simple interpretation of fit as a measure of explained variance but does not consider the number of model parameters.

## Results

### Accounting for Population Genetic Structure With PC Projection in UKBB

We started by defining four different PC adjustment approaches to correct for population genetic structure: 1) PC projection onto the PC space obtained from a subset (*n* = 5,000) of independent samples from the same cohort as the discovery or target set (PC_UKBB_); 2) PC projection onto the PC space obtained from all samples from the 1000 Genomes Project (PC_1KG_); 3) similar to approach 2, but using only European samples (PC_EUR_); 4) similar to approach 2 but using only non-European samples instead (PC_NEU_). For each four above-mentioned PC adjustments, the external sample set was used to infer the eigenvectors of the PC space, then genetic data from discovery or target samples were transformed applying these eigenvectors, with an operation called “projection” (Bycroft et al., 2018).

**FIGURE 1:**
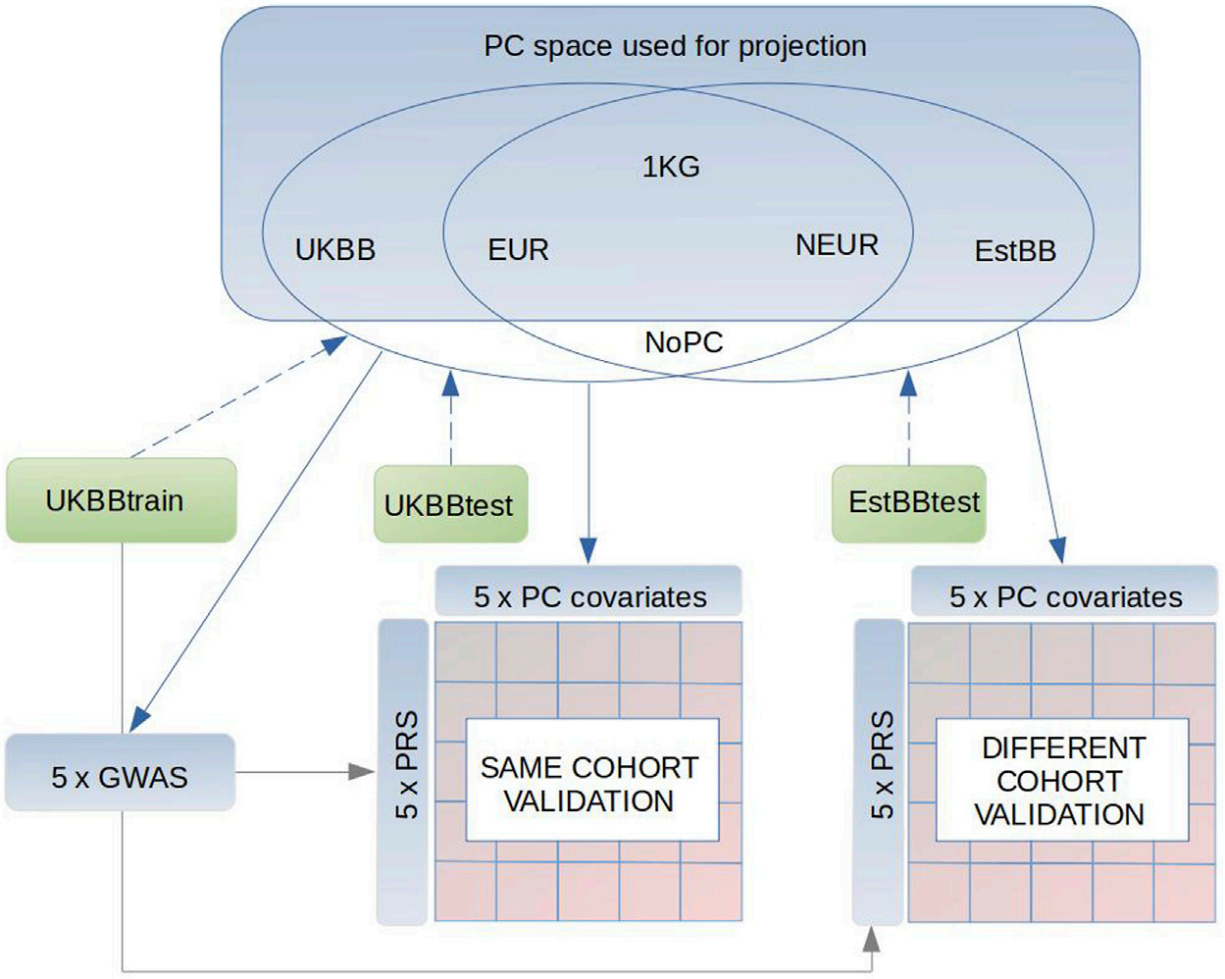
Schematics of our study design. Briefly, we used 1000G as a reference dataset to conduct the PCAs in three subsets (1) only Europeans (EUR), (2) non-Europeans (NEUR), (3) all 1000G samples (1 KG). Also we conducted PCAs in subsets of 5,000 individuals from the UK Biobank (UKBB) and Estonian Biobank (EstBB), which are respectively independent from the UKBBtrain (GWAS sample), UKBBtest and EstBBtest target sets. Following, the UKBBtrain, UKBBtest, EstBBtest were projected in these PC spaces (blue dashed arrow) to receive the PCs (PC1:PC20) to adjust in the GWASs and target sets (blue continuous arrows), where the PRSs performance was tested. As a result of different PC adjustments plus one control (PC_0_) in GWAS and accordingly in both target sets, UKBBtest and EstBBtest, we reached 25 different validation models in both sets. Gray continuous arrow points to the datasets, where the GWAS summary statistics were applied.

We computed the PC coordinates of the discovery and target samples of the UKBB by projecting these samples onto the four different PC spaces (Supplementary Figure S1). Next, we ran four independent GWASs correcting for the first 20 PCs derived from the four different PC spaces described above, and computed PRS relying on summary statistics derived from these association studies. Depending on the PC set used for the GWAS correction, we obtained summary statistics to calculate PRS_UKBB_, PRS_1KG_, PRS_EUR_, and PRS_NEU_ in the independent target set of UKBB samples. As a control, we also used the results from the GWAS without any PC adjustment for both traits to construct a PRS (PRS_0_). Genomic inflation values for each GWAS version have been reported together with the QQ-plots (Supplementary Figure S2a and Supplementary Figure S2b for height and BMI, respectively). We then validated these PRSs applying linear regression in target sets also including sex, age, genotyping batch and one of the four PC sets or no PCs as covariates. As a result of four different PC sets and one model without PC adjustment, we reached 25 independent validation models for height and BMI both. See Figure 1 for a schematics of the study design.

**FIGURE 2:**
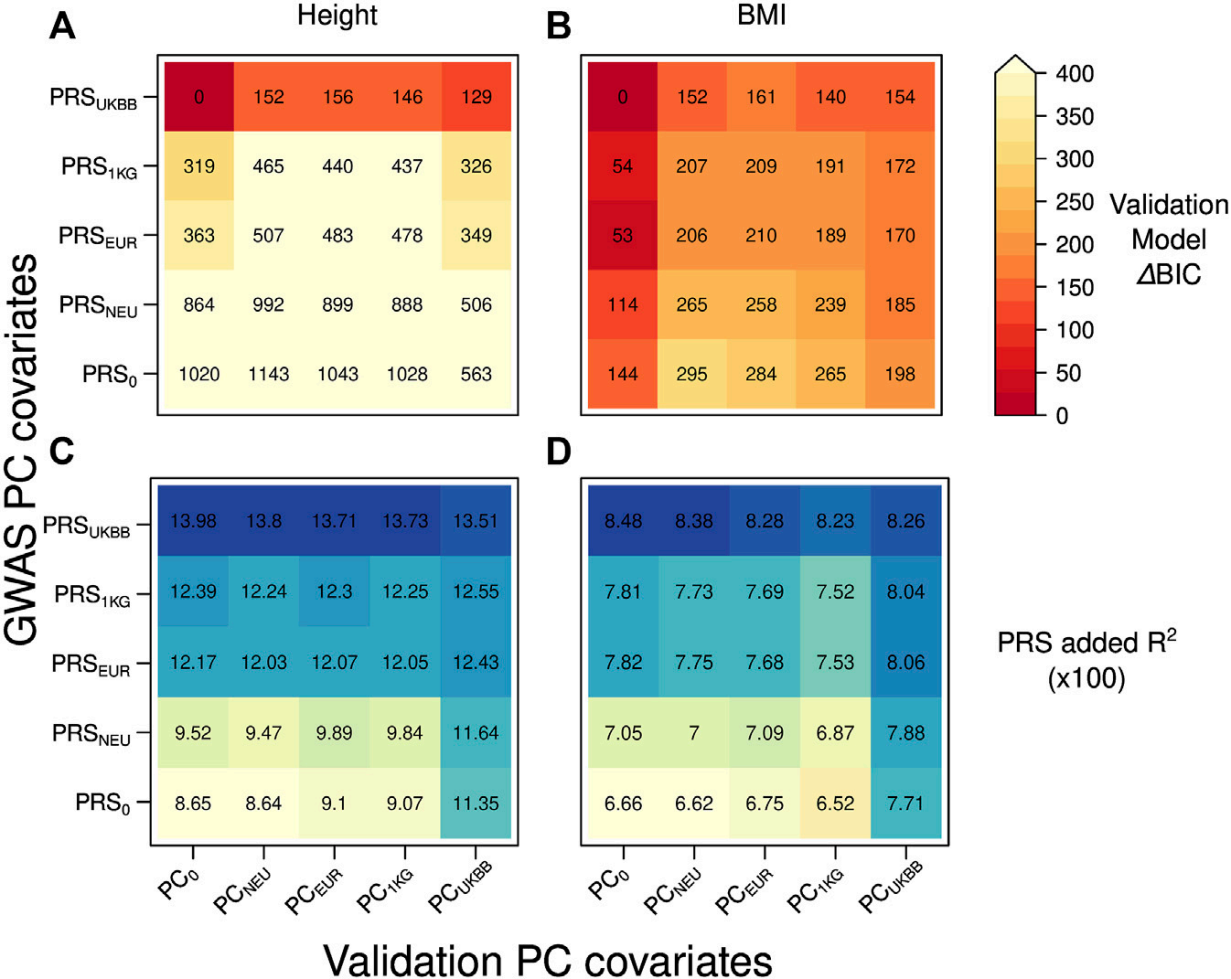
Heatmap reporting ΔBIC values for 25 different validation models in case of the independent discovery (UKBBtrain) and target set (UKBBtest) originating from the same large cohort: **(A)** height **(B)** BMI. For each model, we computed ΔBIC (difference between each model’s BIC value minus BIC for the best-fitting model). The lower ΔBIC value is indicated by darker red color (the lower the ΔBIC value, the better fit the validation model is). Heatmap with the added R^2^ values by the PRS for 25 different validation models in case of the independent discovery (UKBBtrain) and target set (UKBBtest) originating from the same large cohort: **(C)** height **(D)** BMI. A higher R^2^ is indicated with a darker blue color. Y-axis: five GWASs conducted in UKBBtrain, which summary statistics were applied for PRSs calculations used in the validation models of target set. These PRSs were then used in a validation model also adjusted for age, sex, genotyping batch, and 20 first principal components from four different PCAs for UKBBtest plus one validation model without any PC adjustment as a control (x-axis).

We compared the model fit by their BIC values and by the added *R* ^2^, the amount of variance explained by PRS in each validation model, received by subtracting from the model’s total *R* ^2^ the one obtained without PRS, as shown in Figure 2. To see the relative difference in the fit of the validation models, we reported ΔBIC values (difference between each model’s BIC value and the BIC of the best-fitting model) when predicting height and BMI in Figures 2A,B, respectively. The model with smallest BIC value for both height and BMI contained the PRS based on the summary statistics received from GWAS adjusted for the dataset dependent PCs resulting in PRS_UKBB_ and no inclusion of PCs as covariates. The validation models containing PRS_0_, that is, the PRS built from GWAS summary statistics that were not corrected for PCs, provided the worst fit to the data (Figure 2A, ΔBIC = 563–1143) when predicting height. PRSs obtained from GWAS summary statistics adjusted for PCs from an external reference set clearly yielded a lower model fit than PRS_UKBB_ (Figure 2A, ΔBIC = 319–992 for the PCs from an external set). This trend can be explained by a less rigorous correction of population structure offered by the externally derived PCs during GWAS, which is most severe for the PRS_NEU_ (ΔBIC = 506–992).

For BMI, besides having the same best-fitting validation model as for height (Figure 2B, PRS_UKBB_ combined with PC_0_), the combinations of any PRSs with no PC adjustment in the validation model lead to smaller BIC values (Figure 2B, ΔBIC = 0–144). While all validation models including PCs as covariates provide larger BIC values (ΔBIC = 152–295), PC_UKBB_ seems to perform better than any other PC adjustment (ΔBIC = 154–198).

As expected, when looking at the added *R* ^2^ by PRS (Figures 2C,D), the best performance was obtained by PRS_UKBB_ both when predicting height and BMI (13.98–13.51% and 8.48–8.23%, respectively), irrespective of the PC set chosen as covariates. While this results underlines the inadequacy of projected PCs in accounting for population stratification during GWAS, it also shows that the residual confounding effect decreases PRS predictivity when validating it in a separated sample set, even within the same cohort. Notably, the sharp decrease in added *R* ^2^ shown by other PRSs (the lowest R^2^ value of 8.64% for height in case of PRS_0_-PC_NEU_) is less extreme when including dataset-specific PCs during validation (11.35% for PRS_0_-PC_UKBB_). This can be due to a mild case of Simpson’s paradox (Wagner, 1982), where projected PCs (or no PCs at all) are unable to resolve the population stratification during PRS validation, causing a loss of PRS predictivity (see Supplementary Figure S3). Nevertheless, when focusing on PRS_UKBB_, we observe a decrease in added *R* ^2^ when using PC_UKBB_, a sign that indeed residual population stratification might be present also in what is considered the golden standard. To further investigate the correlations between PRS, PCs, and predicted trait, we focused on PC_UKBB_, which provided the highest explained variance during validation for both traits (Supplementary Figure S4a-4d, last column) and tested its correlation with other covariates. Population structure summarized by the first 20 PCs did indeed explain some variance in height (1.4%, see Supplementary Table S1), but not in BMI (F-test, *p* = 0.012 at the Bonferroni corrected *p*-value threshold of 0.005). However, these PCs still explained a significant proportion of PRS variance (2.4% for height PRS_UKBB_ and 1.7% for BMI PRS_UKBB_), even though the underlying GWAS and validation model both were corrected for the same PCs (PC_UKBB_). A reason for small but very significant (*p* = 1.46E-25 for height) PCs and PRS correlations could be an incomplete correction for population structure at each locus, a possibility explored by Zaidi and Mathieson (2020), which is amplified by summing single SNP effect sizes as done in PRS construction. Indeed, when correcting GWAS for PCs resulting from projection on an external reference population or performing no correction at all, the resulting PRS consistently showed much stronger correlation (e.g., shown by 49.4% PRS_0_, 20.0% PRS_1KG_, 21.6% PRS_EUR_, 43.0% PRS_NEU_ explained variance for height) with population structure (PC_UKBB_) in the target set. Notably, height PRSs demonstrated higher correlations with population structure than BMI PRSs across the board.

When predicting height, the incomplete correction of PRS for population structure results in a portion of explained variance shared by PRS and PCs. When firstly regressing out the effect of PCs on the trait, the trait variance explained only by PRS_UKBB_ is lower than when predicting the trait unadjusted for PCs by PRS_UKBB_ (−1.2% in trait_res_PCs ∼ PRS_UKBB_ vs. trait_res ∼ PRS_UKBB_, Supplementary Table S1). These differences are all higher for poorly corrected PRSs (−4.5–5.7% for PRS_0_ or any PRS received based on an external reference set). Likewise, when the effect of PRS on the trait is first regressed out, the trait-PCs correlation is lower than simple PC-explained trait variance (−0.7% for trait_res_PRS_UKBB_ ∼ PCs vs. for trait_res ∼ PCs). *R* ^2^ and F-test *p*-values for the tested regressions are shown in Supplementary Table S1.

### PC Correction for a Target Set From a Population Other Than the Discovery One

**FIGURE 3:**
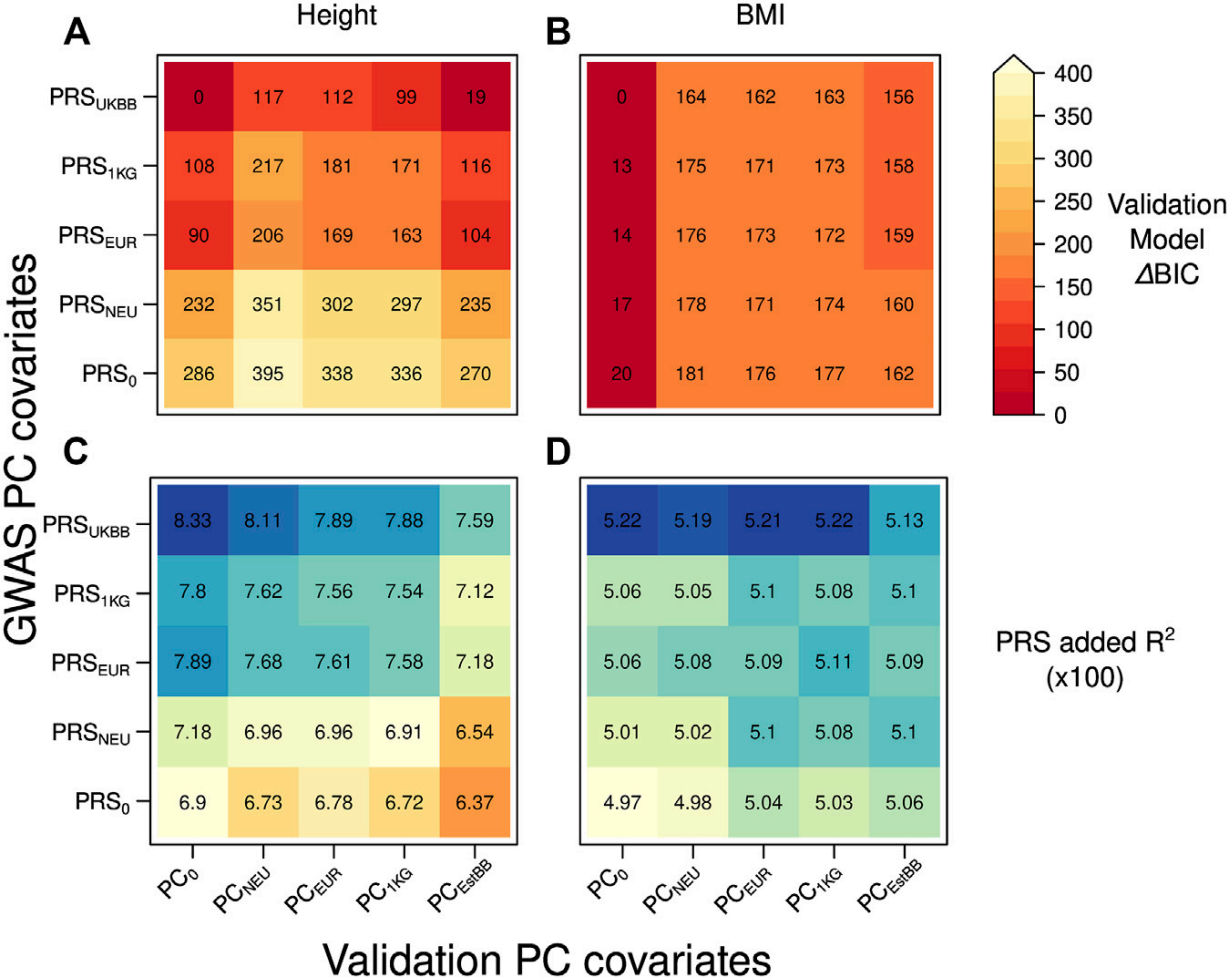
Heatmap with the ΔBIC values for 25 different validation models in case of the discovery (UKBBtrain) and target set (EstBBtest) originating from the different cohort: **(A)** height **(B)** BMI. For each model, we computed ΔBIC (difference between each model’s BIC value minus BIC for the best-fitting model). The lower ΔBIC value is indicated by darker red color (the lower the ΔBIC value, the better fit the validation model is). Heatmap with the added R2 values by the PRS for 25 different validation models in case of the discovery (UKBBtrain) and target set (EstBBtest) originating from the different cohort: **(C)** height **(D)** BMI. Y-axis: five GWASs conducted in UKBBtrain, which summary statistics were applied for PRSs calculations used in the validation models of target set. These PRSs were then used in a validation model also adjusted for age, sex, genotyping batch, and 20 first principal components from four different PCAs for EstBBtest plus one validation model without any PC adjustment as a control (x-axis).

To test whether the projection on an external dataset improves the PRS transferability in a different target cohort, we used as validation set the data from the EstBB applying the same PC corrections described for the UKBB target set, except for PC_EstBB_ being computed onto PCA of 5,000 EstBB instead of 5000 UKBB samples (Figure 3).

When moving to a different European cohort, similar PCs-PRS-trait correlation patterns were observed as in case of the same-cohort discovery and target set. The dependency of trait and PRS_EstBB_ on population structure (presented for PC_EstBB_ only) was comparable to the ones in the UKBBtest set (Supplementary Tables S1), except for the height-PC_EstBB_ correlation being stronger (3.4%). Similarly to the UKBB target set, in the EstBB set, the height-PC_EstBB_ correlations were consistently stronger than for BMI-PC_EstBB_, which shows that BMI is again less dependent on population structure. However, differently from the scenario of testing in the same cohort, a poor or absent PC correction in GWAS (PRS_0_) did not yield a PRS that was highly correlated with population structure (PC_EstBB_), although a small increase is still visible compared to the PRS_UKBB_ (e.g., for height 3.1% PRS_UKBB_ vs. 4.2% PRS_0_, 4.0% PRS_1KG_, 4.9% PRS_EUR_, 4.2% PRS_NEU_).

Nevertheless, similar to the scenario of having the discovery and target set from the same cohort, we now found that when predicting a trait, the best-fitting model according to BIC value was the one with PRS computed by applying summary statistics from the GWAS adjusted for the dataset dependent PCs (PRS_UKBB_) and no PC (PC_0_) adjustment during PRS validation (Figures 3A,B for height and BMI, respectively). The closest performance to the best-fitting models was consistently shown by the models containing PRS_UKBB_ together with any possible PC adjustment in the validation model for height (Figure 3A, ΔBIC = 0–117), although PRS_1KG_ and PRS_EUR_ were better than in the same-cohort validation. For BMI, the lowest ΔBIC values were demonstrated by the validation models without PC covariate (PC_0_) combined with any PRS (Figure 3B, ΔBIC = 0–20). Similar to the first scenario, when looking at added *R* ^2^ of the various models (Figures 3C,D for height and BMI, respectively), we observe a slight decrease in validation models including PCs (Figures 3B,C, columns two to five), pointing to a residual presence of population structure in the PRS.

As the datasets to conduct PCAs vary in size (*n* = 503 for 1000G EUR subset up to 5,000 for the PC_UKBB_ and PC_EstBB_), we also computed ΔBIC, added *R* ^2^ and total *R* ^2^ values using a fixed size (*n* = 500) for the samples used to compute the PCA and onto which the remaining samples were projected (Supplementary Figures S5–7), and found this to not alter our results in a qualitative way. Also, the correlations between the original PCs received based on different size PCA approaches versus fixed size (*n* = 500) in UKBBtest and EstBBtest sets are provided in the Supplementary Tables S2a-d and Supplementary Tables S3a-d, respectively. We also computed PCs controlling for shrinkage to mitigate potential issues emerging during the projection process, as well as computing principal axes of genetic variation starting from a matrix of identity by descent (IBD) distances. While the PCs received after controlling for shrinkage were comparable to the ones obtained without (Supplementary Tables S4a,b), the IBD-based analyses (Supplementary Tables S5a-d and Supplementary Tables S6a-d, respective to the target set) showed that such an approach could leverage on a finer level of population structure which, however, is beyond the scope of the current work aimed at exploring best practices when using methods controlling for population structure described by common variants.

## Discussion

To test whether adjusting GWAS for the PCs received via the projection approach would improve the PRS model fit in a target set from a different cohort and whether the PC adjustment in the validation model is needed, we performed various sets of PC corrections in GWASs and in validation models of corresponding PRSs.

For height, the added *R* ^2^ of the best-fitting validation model explained 13.98% and for BMI, 8.48% of the total variance in the UKBBtest target set. We confirmed that the cohort-specific PCs in GWAS yield a better performing PRS (PRS_UKBB_) in a target set from the same population than the PCs calculated by projecting the GWAS samples into the reference dataset of 1000G. Such a reduction is not counterbalanced by an improvement in transferability to a different cohort than the one from which summary statistics were obtained, as shown when computing PRS based on the UKBB GWAS for the individuals from EstBB.

Resorting to a cohort-specific PC adjustment (PC_UKBB_ or PC_EstBB_) as the best and most sensible approach in GWAS and PRS validation, we elaborated on the implications of PCs inclusion in the validation model. When purely considering model fitness, adding PCs would be worthless for a trait that does not show any correlation with population structure, such as BMI, since they do not add explanatory power while increasing the number of covariates, but in principle, they would be constructive for structured traits, such as height. The observation that also for height the lowest BIC values for our validation models were obtained when no PC adjustment was applied points to a residual presence of population stratification in the computed PRS, showing its capacity to represent both true biologically related and spurious population structure information simultaneously. This indication is further confirmed by the slight decrease in added *R* ^2^ when PCs are indeed included as covariates in the validation models of both UKBB and EstBB. Doubts over the efficacy of PCs adjustment have been reported also in previous studies (Haworth et al., 2019; Zaidi and Mathieson, 2020). Indeed, we show that PRSs contain information about population structure even when PC-corrected, and even for traits which appear non-structured (BMI). Therefore, even if BIC would warrant the exclusion of PCs in a model selection scope, they should be included when predicting a structured trait (height), to account for the residual population structure confounding effect in PRS and correctly evaluate its added predictive value. Conversely, even if PRSs for ideal non-structured traits also contain information about population structure, the latter cannot operate as a confounder: in this case, PCs inclusion in the validation model does not have any clear utility or consequence. Since testing the correlation between PCs and the target trait is computationally inexpensive, we recommend this as a preliminary check to inform the user about the need to include PCs in the prediction model.

The same conclusion drawn for the UKBB results holds when the discovery and target sets originated from different cohorts. The added *R* ^2^ of the validation model in EstBB computed using summary statistics from UKBB explained, respectively, 8.33 and 5.22% of the total variance in height and BMI in the target set. We acknowledge that besides the differences in the genetic settings for UKBB and EstBB datasets, the cohorts diverge in age range and sex proportions, and these could also influence the results. Indeed, it has been shown that even among the same ancestry group, the PRS prediction accuracy can vary due to differences in the discovery and target sets’ age, sex or socioeconomic distribution (Mostafavi et al., 2020).

Furthermore, we did not detect very large numeric differences in the total explained variance by the validation models containing PRSs and PCs received via projection onto different sets of external reference data. Firstly, it could be that none of these sets reflected the population structure of our study sample well. That argument was supported by observing smaller correlations between the PRS and PCs, when we used the GWAS summary statistics adjusted for the dataset-dependent PCs (PC_UKBB_) for the PRS calculations. Additionally, such small differences could occur since for each validation model we allowed the PRSice software to choose the PRS with the highest *R* ^2^ value, which means that PRSs in different validation models could contain different numbers of SNPs. On one hand, by choosing the best-performing PRS for each validation model, we might unintentionally diminish the possible differences caused by four different PC adjustments for GWASs reflected on the effect sizes differences for each individual associated SNP. On the other hand, choosing the same associated SNPs for each PRS calculation would limit the prediction accuracy of the validation model. Also, a minor caveat is that the reported added *R* ^2^ were estimated in-sample; however, the small parameter space explored during PRS optimization (PRS effect size and eight different *p*-value thresholds) decreases the risk of over-fitting.

Given the clinical potential of PRSs, it is of utmost importance to explore the methods to adjust for population genetic structure resulting in less biased predictions and making personalized medicine more accessible for everyone. Here, we found that the best-fitting validation models for height and BMI both did not contain any genetic PCs and it included the PRS applying the summary statistics from the GWAS adjusted for the dataset-dependent PCs. This finding was similar for UKBB and EstBB as a target set, showing that projecting on an external reference set does not improve its transferability. Furthermore, although dataset-dependent PC correction during GWAS is the best approach among the ones we tested, our results confirm that, while reducing it, cannot prevent residual population structure information into PRS, which may or may not exert a confounding effect depending on the trait’s genuine link to population structure. Finally, we found no evidence pointing against the usage of dataset-specific PCs also during validation. Therefore, even though their implications should be carefully evaluated depending on the PRS, trait and PCs actual correlations, PC covariates should be conservatively added in the validation model.

## Data Availability

The data analyzed in this study are subject to the following licenses/restrictions: The data that support the findings of this study are available through the original publications and repositories: data from UK Biobank at https://biobank.ndph.ox.ac.uk/showcase/ (accessed under Project #17085); data from Estonian Biobank at https://genomics.ut.ee/en/access-biobank (accessed with Approval Number 285/T-13 obtained on 17/09/2018 by the University of Tartu Ethics Committee). Requests to access these datasets should be directed to https://biobank.ndph.ox.ac.uk/showcase/; https://genomics.ut.ee/en/content/estonian-biobank.
