# Supplementary material for: A Principal Component Informed Approach to Address Polygenic Risk Score Transferability Across European Cohorts
Source: Front Genet. 2022 Jul 18;13:899523. doi: 10.3389/fgene.2022.899523 (PMC9340200; doi:10.3389/fgene.2022.899523)
Supplement: Supplementary file 1 [file DataSheet1.PDF]

Supplementary Figures

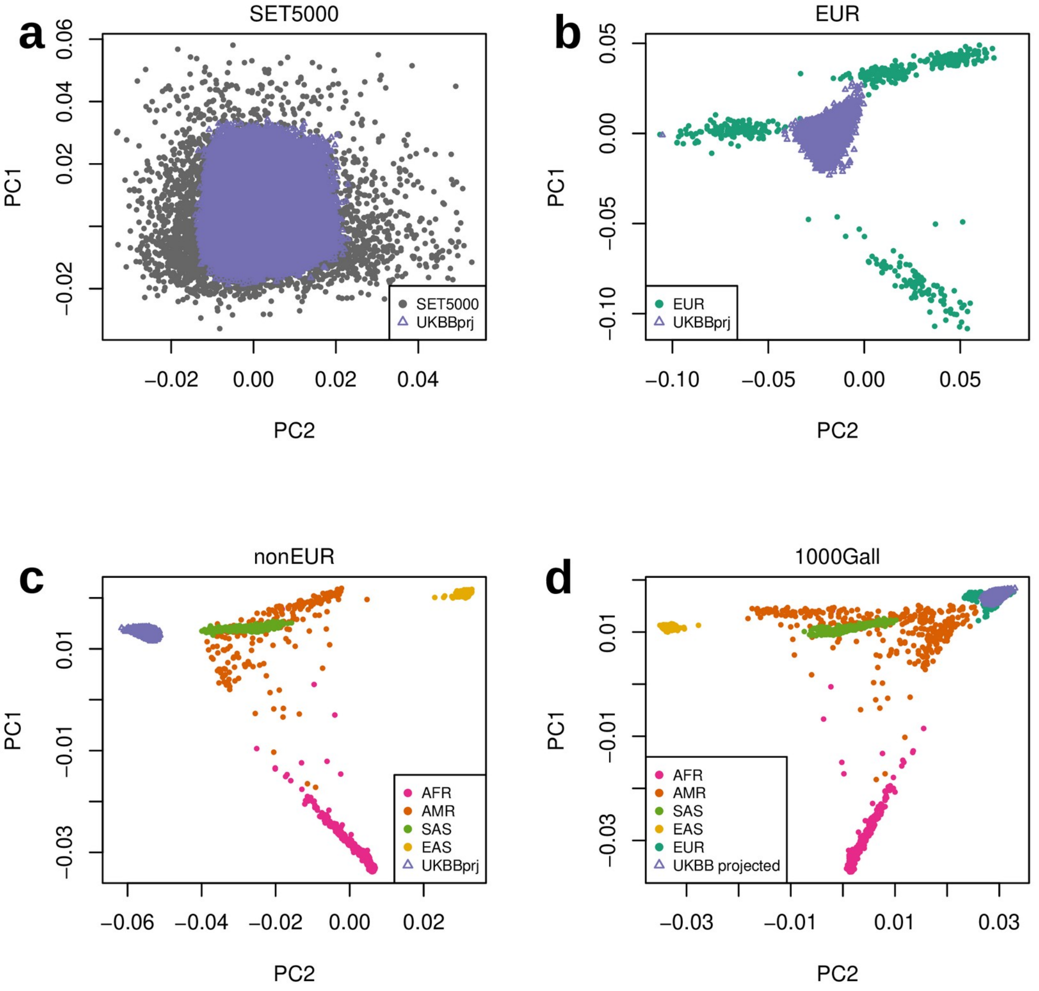

Supplementary Figure 1. Four different versions of PC spaces in the discovery set: (a) PC_{UKBB}, (b) PC_{EUR}, (c) PC_{NEUR}, (d) PC_{1KG}. These PC spaces were used to derive principal components to adjust for in GWASs by projecting the discovery sample (UKBBtrain) individuals onto these. Each symbol (dot or a triangle) on the PCA plot represents genetic coordinates of one individual. Dots mark the sub-populations, from which the genetic data were used to infer the eigenvectors and triangles mark the samples, whose genetic data were not used to infer the eigenvectors but were instead projected onto the PC space. For illustrative purposes we have presented the first two principal components (PC1 and PC2, marked respectively on x and y-axis) to describe the genetic variation in the discovery sample.

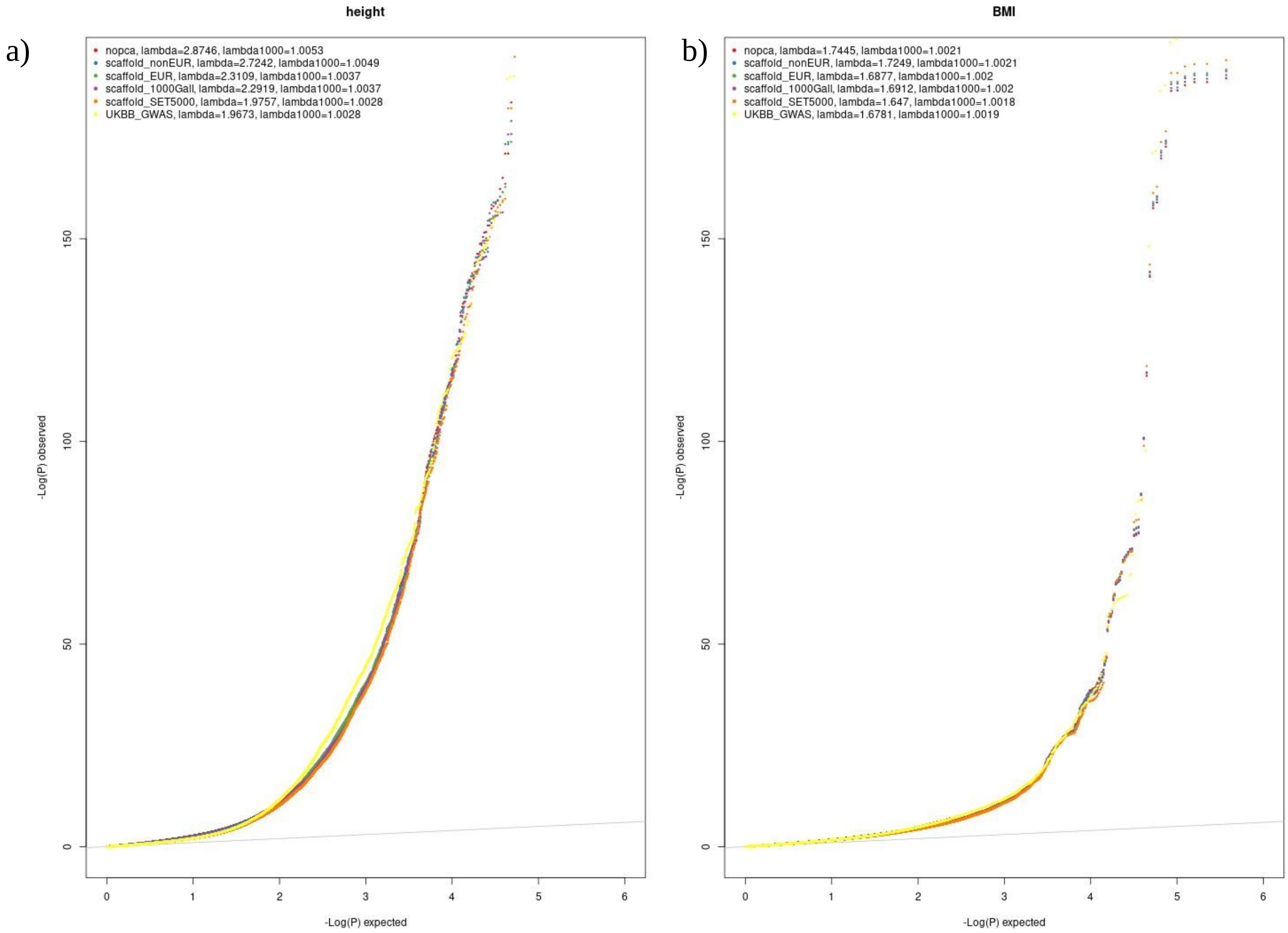

Supplementary Figure 2. Lambda and lambda1000 reported for five GWASs conducted: a) for height and b) for BMI in the UKBBtrain set. As a reference (not used in the current study), the lambda and lambda1000 were calculated for the GWAS for height and BMI, respectively, conducted by the Neale Lab: http://www.nealelab.is/uk-biobank (marked with yellow colour).

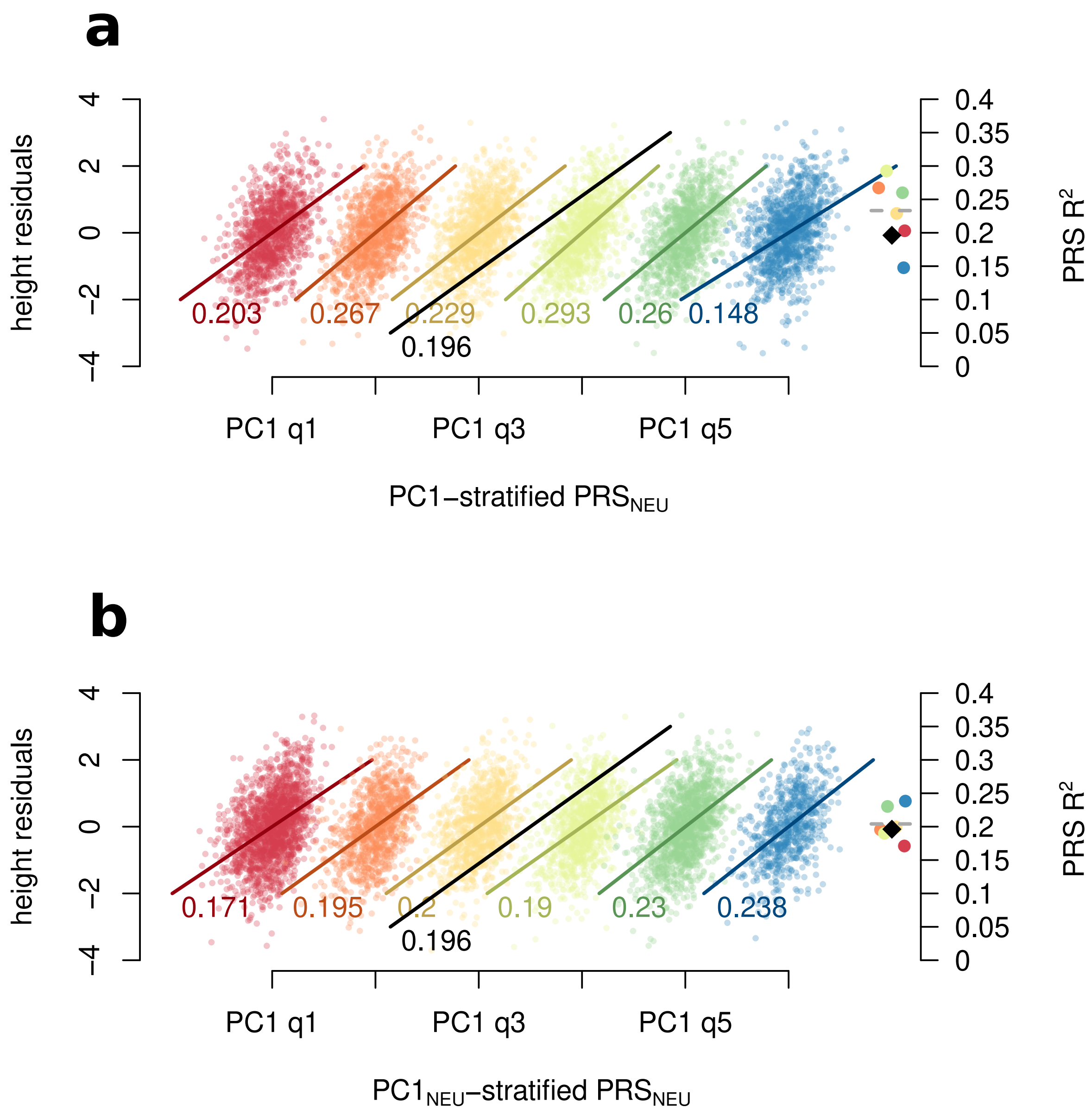

Supplementary Figure 3. PRS_NEU predictivity is lower than expected when correcting with projected PCs, or no PCs. Here we show the trend lines when regressing height residuals after correcting for non-genetic covariates on PRS_NEU, both for the full sample set (black line) and when stratifying in six PC1 quantiles (coloured lines and dots). On the right side we show the realized R^2 for the whole dataset (black diamond) and for the separated quantiles, with their average (grey dashed line). (a) When stratifying for PC1_UKBB we see that the correlation in all groups is, on average, higher than when all groups are combined. (b) This does not happen when PC1 is derived from PC_NEU, showing that this PC space is not able to resolve enough the population structure to make the actual PRS-trait relationship visible.

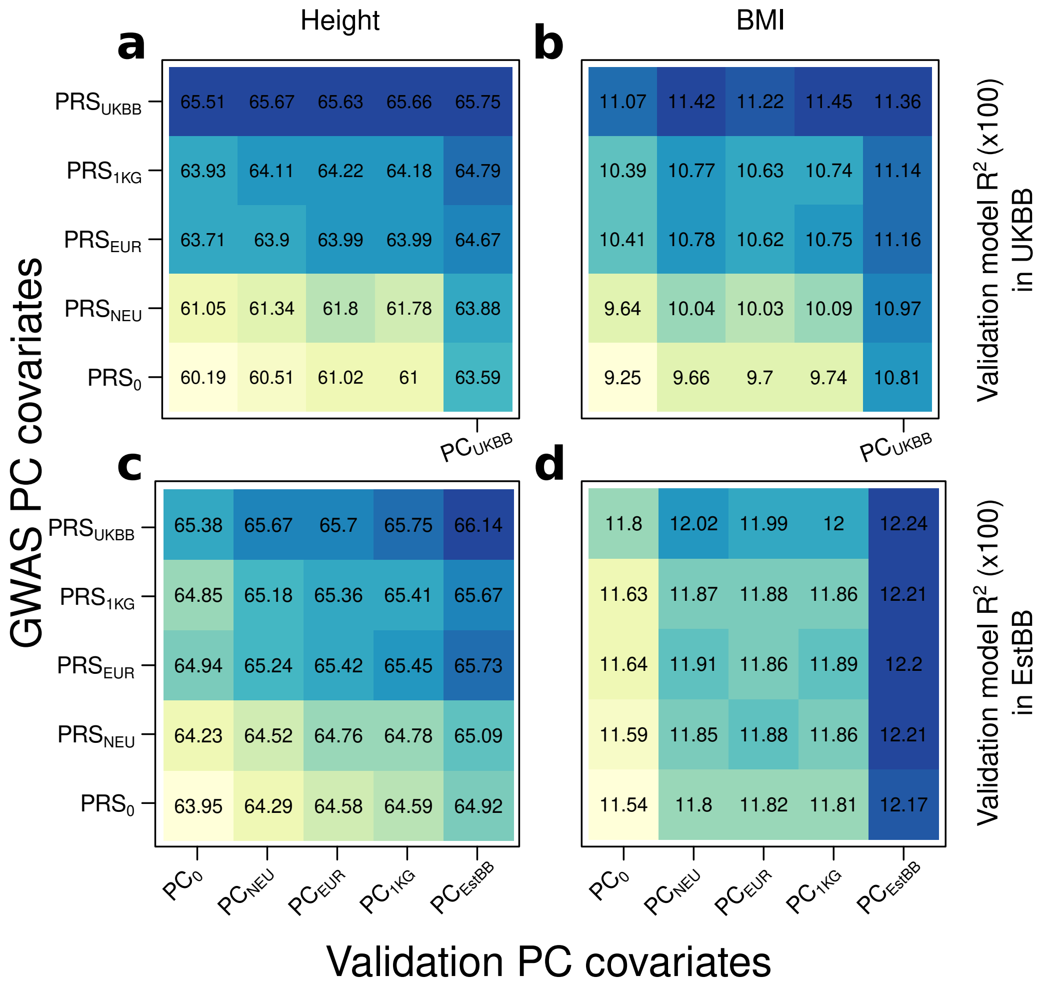

Supplementary Figure 4. The total R^2 values for the validation models in the UKBB target set for a) height and b) BMI, and in the EstBB target set for c) height and d) BMI, respectively. Y-axis: five GWASs conducted in UKBBtrain, which summary statistics were applied for PRSs calculations used in the validation models of target set. These PRSs were then used in a validation model also adjusted for age, sex, genotyping batch, and 20 first principal components from four different PCAs for UKBBtest plus one validation model without any PC adjustment as a control (x-axis).

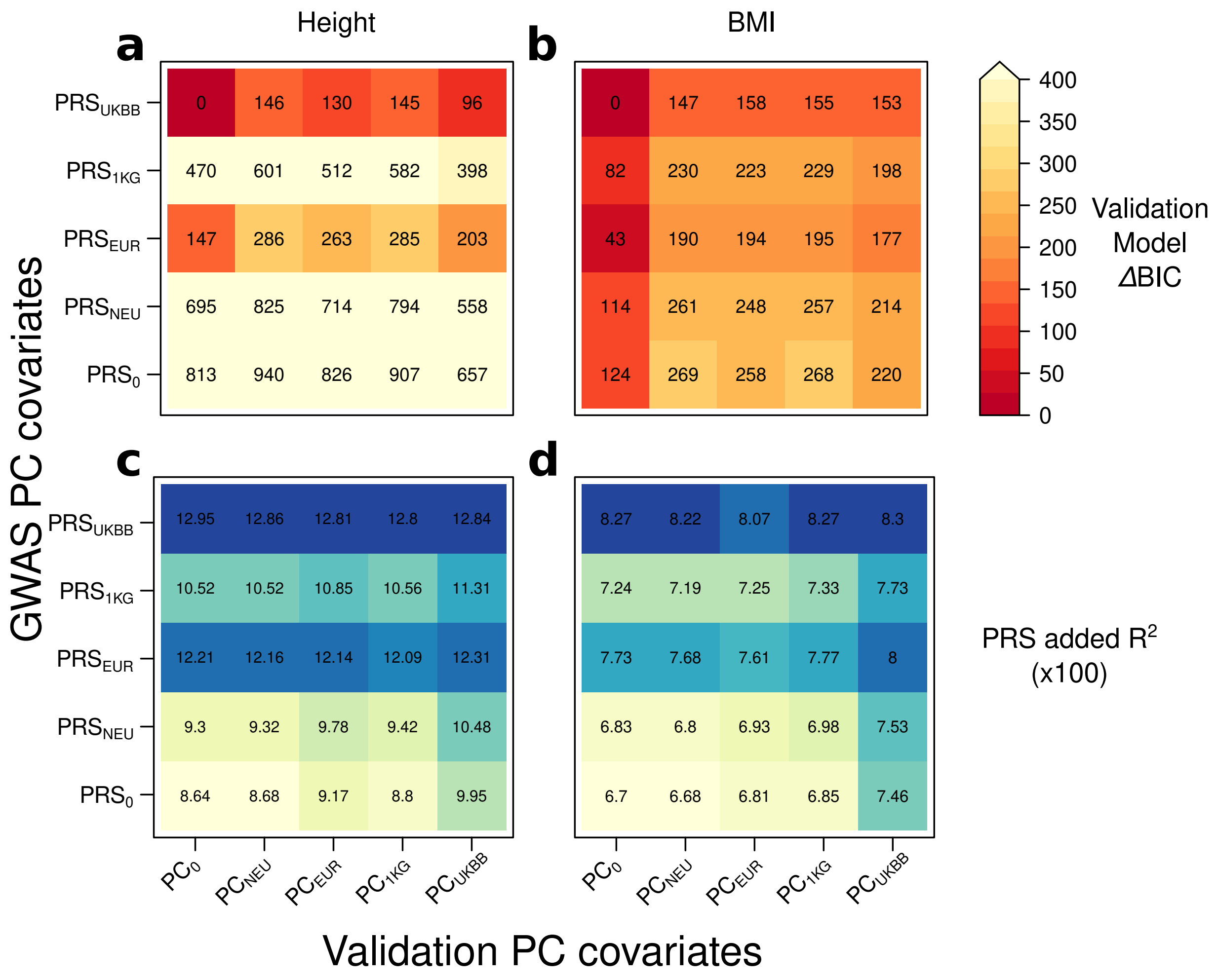

Supplementary Figure 5. The ΔBIC values for the validation models of a) height and b) BMI in the UKBB target set, whereas the PCAs to receive the PCs for the discovery and target set were conducted on fixed sample size of 500 individuals for the comparability purposes. Y-axis: five GWASs conducted in UKBBtrain, which summary statistics were applied for PRSs calculations used in the validation models of UKBB target set. These PRSs were then used in a validation model also adjusted for age, sex, genotyping batch, and 20 first principal components from four different PCAs for UKBBtest plus one validation model without any PC adjustment as a control (x-axis). Added R^2 values for the PRSs for c) height and d) BMI in the validation models in the same UKBB target set, whereas the PCAs to receive the PCs for the discovery and target set were conducted on fixed sample size of 500 individuals for the comparability purposes.

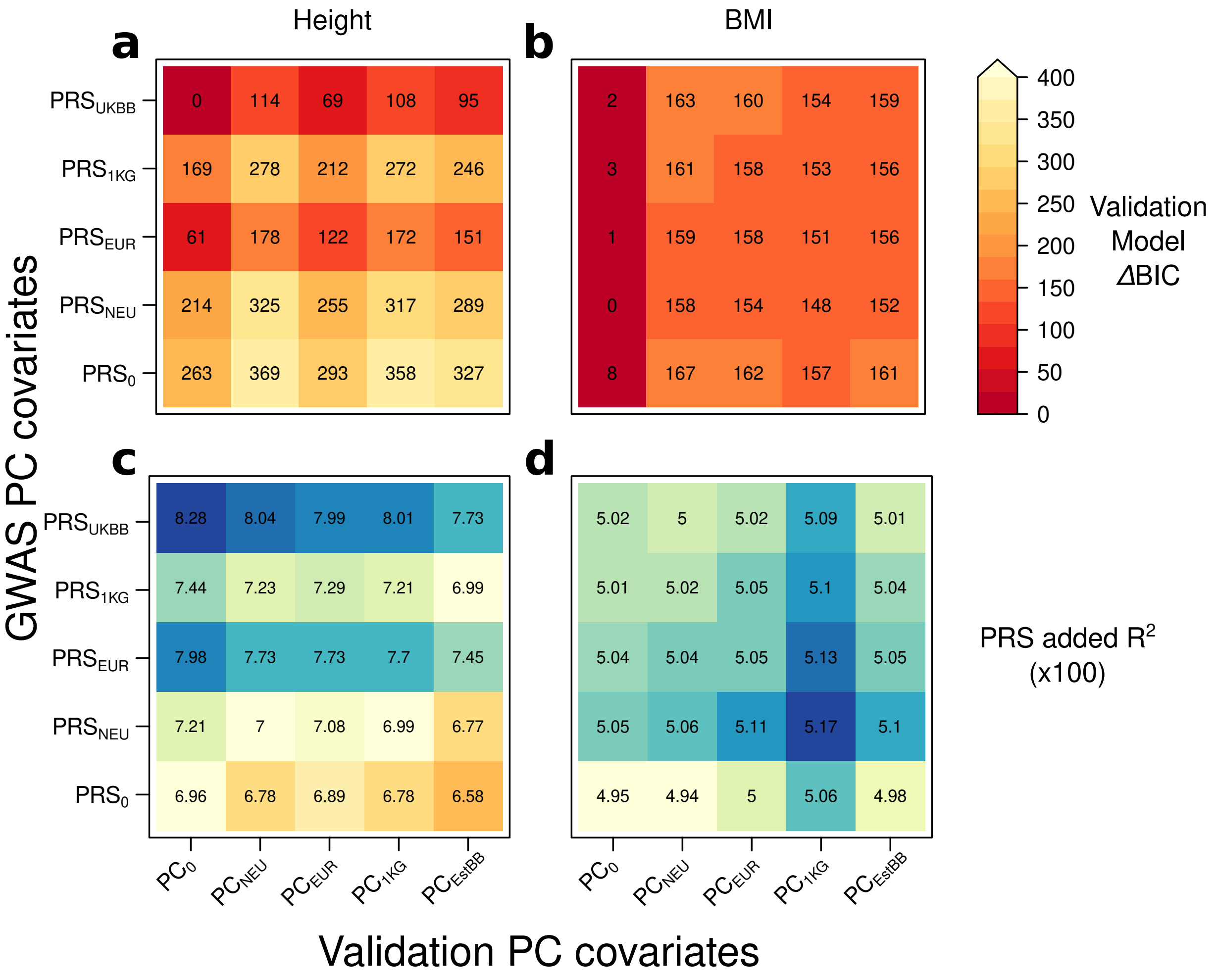

Supplementary Figure 6. The ΔBIC values for the validation models of a) height and b) BMI in the EstBB target set, whereas the PCAs to receive the PCs for the discovery and target set were conducted on fixed sample size of 500 individuals for the comparability purposes. Y-axis: five GWASs conducted in UKBBtrain, which summary statistics were applied for PRSs calculations used in the validation models of EstBB target set. These PRSs were then used in a validation model also adjusted for age, sex, genotyping batch, and 20 first principal components from four different PCAs for EstBBtest plus one validation model without any PC adjustment as a control (x-axis). Added R^2 values for the PRSs for c) height and d) BMI in the validation models in the same EstBB target set, whereas the PCAs to receive the PCs for the discovery and target set were conducted on fixed sample size of 500 individuals for the comparability purposes.

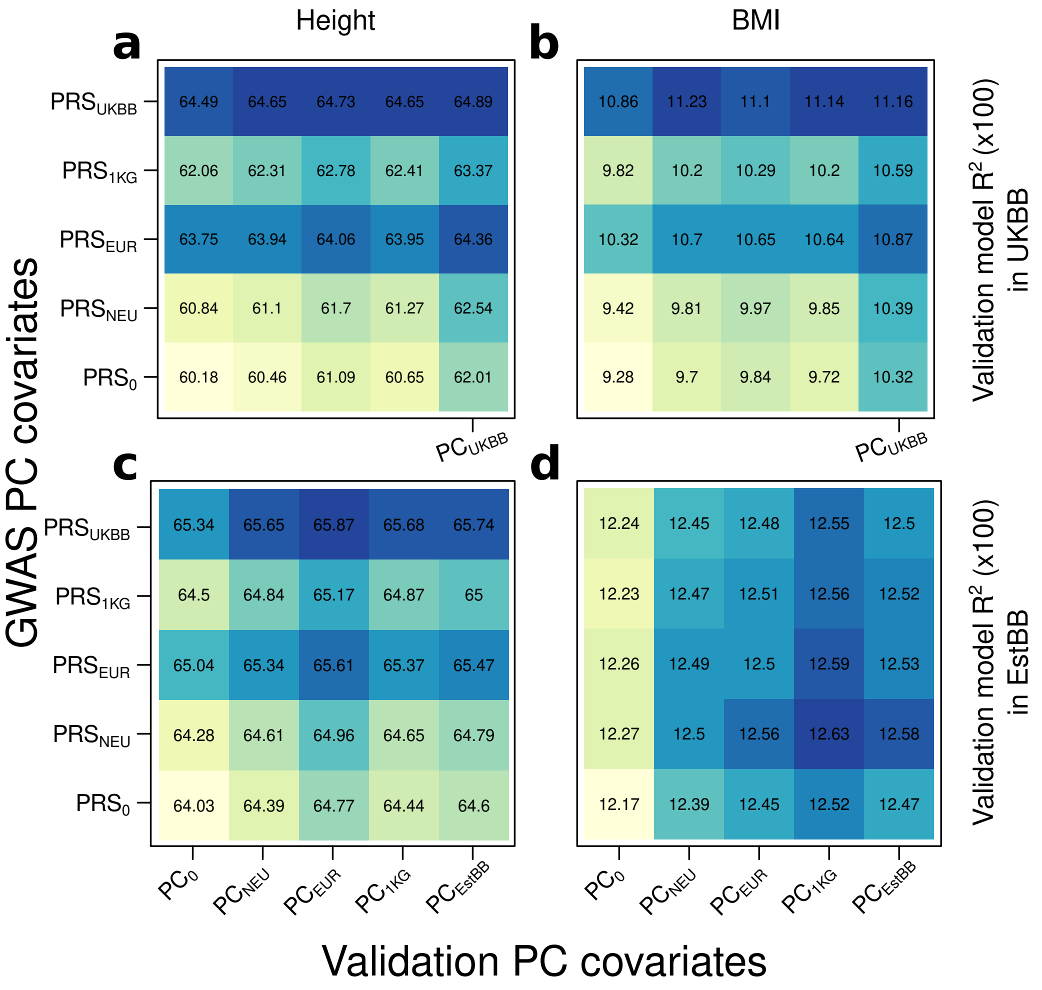

Supplementary Figure 7. The total R^2 values for the validation models in the UKBB target set for a) height and b) BMI, and in the EstBB target set for c) height and d) BMI, respectively, whereas the PCAs to receive the PCs for the discovery and target sets were conducted on fixed sample size of 500 individuals for the comparability purposes. Y-axis: five GWASs conducted in UKBBtrain, which summary statistics were applied for PRSs calculations used in the validation models of target set. These PRSs were then used in a validation model also adjusted for age, sex, genotyping batch, and 20 first principal components from four different PCAs for the UKBBtest and for the EstBBtest target sets plus one validation model without any PC adjustment as a control (x-axis).
